# Skin-Related Sexual Life Questionnaire (SRSLQ): Creation and Validation of the Questionnaire

**DOI:** 10.3390/medicina59112023

**Published:** 2023-11-17

**Authors:** Kamila Kędra, Adam Reich

**Affiliations:** 1 Department of Dermatology, Institute of Medical Sciences, Medical College of Rzeszow University, 35-055 Rzeszow, Poland; kedra.kamilaa@gmail.com; 2 Doctoral School, University of Rzeszow, 35-959 Rzeszow, Poland

**Keywords:** stigmatization, skin, psoriasis, sexual dysfunction, quality of life

## Abstract

*Background and Objectives*: The assessment of sexual dysfunction among psoriatic patients still remains a great diagnostic difficulty due to its subjective and embarrassing character. Various questionnaires have been used to assess sexual dysfunctions associated with skin conditions; however, none of them have been approved as a gold standard. The aim of our study was to create and validate the Skin-Related Sexual Life Questionnaire (SRSLQ) for the assessment of possible sexual dysfunction and related psychological burdens in patients with skin diseases. *Materials and Methods*: A total of 109 patients, including 45 women and 64 men (1:1.4) suffering from psoriasis and aged between 18 and 73 years (mean 48.0 ± 13.4 years), were included in the study. All subjects completed the 11-item SRSLQ and the Dermatology Life Quality Index (DLQI). In addition, all men were asked to fulfil the five-item version of the International Index of Erectile Dysfunction (IIEF-5) at the time of examination and 7 days after enrollment. The disease severity was assessed via the PASI scale. *Results*: The statistical analysis of internal consistency of SRSLQ showed very good integrity, with a Cronbach α coefficient of 0.94. The reproducibility level assessed with intraclass correlation coefficient (ICC) amounted to 0.96. A significant correlation between the total score of the 11-item SRSDQ and the DLQI (r = 0.39; *p* < 0.001) was found, confirming congruent validity. *Conclusions*: The validated 11-item Skin-Related Sexual Life Questionnaire can be successfully implemented in daily clinical practice as well as in clinical research.

## 1. Introduction

Psoriasis is a classic example of a common, chronic skin condition with a debilitating influence on a patient’s well-being. It is triggered by genetic, environmental, and psychological factors, with an estimated prevalence of 2–4% in Western countries [1]. It can manifest itself as a wide spectrum of skin lesions of varying severity, which may include single psoriatic plaques located over the elbows and knees or may cover almost the entire body surface, including such locations as the face, hands, and genital area. The involvement of the latter areas is often described as particularly embarrassing and stigmatizing [2,3,4]. According to the studies, the occurrence of psoriatic lesions in the genital area at any time during disease, identified by medical professionals during examination or self-reported by patients through surveys, varied between 33% and 63% [5].

As external appearance is one of the major components that is taken into account in interpersonal relationships, psoriasis can lead to a significant decrease in self-esteem and well-being [6,7]. A constant feeling of stigmatization, low self-confidence, and embarrassment may influence the avoidance of starting a family or having children due to the fear of offspring inheriting the disease [8]. In previously conducted studies, it was shown that psoriasis has been associated with numerous psychological disorders, such as depression, anxiety, or suicidal ideation [9]. Gupta et al. described an increase in psoriasis flare-ups and pruritus severity as corelating with an increase in stress and depressive symptoms. An analysis of 27 studies showed a 1.5-times-increased risk of depression and anxiety symptoms among psoriatic patients compared to individuals without psoriasis. Moreover, not only can psoriasis lead to depression; depression may also cause psoriasis due to neurochemical and immunological mechanisms [9,10].

Last but not least, psoriasis may exert a significant impact on the sexual dimensions of patient’s life [11]. Based on previously published studies, sexual dysfunction is observed in 22.6% to 71.3% patients with psoriasis, depending on the assessment method employed [12]. Moreover, a higher risk of erectile dysfunction in psoriatic males compared to healthy controls was observed in five out of eight studies [13]. Similarly, sexual dysfunction was noted in almost half of British women with psoriasis [14]. These disorders require further research in order to better understand their pathogenesis, as well as the scale and consequences of their occurrence. So far, the presence of sexual disorders among psoriatic patients has been associated with the location of psoriatic lesions, showing a positive correlation with certain locations such as the hands, face, and genital area [15]. Another factor which was suggested as having an impact on the occurrence of sexual disorders is the severity of psoriasis; i.e., a higher amount of sexual dysfunction was observed in patients with severe psoriasis when compared to those with milder or moderate forms [16]. Moreover, the elevated prevalence of sexual dysfunction in psoriatic patients may be attributed to sometimes painful and ashaming manifestations of psoriasis, such as stinging, bleeding, and desquamation. Thus, the feeling of discomfort and loss of self-confidence may ultimately impact sexual well-being and function in affected individuals [17]. Other factors may also influence the occurrence of sexual disorders, such as decreased self-esteem, exacerbation of psoriasis affecting appearance, low libido, and discomfort arising from desquamation or use of topical treatment. We also have to bear in mind that patients with psoriasis often suffer from a number of comorbidities such as dyslipidemia, diabetes, hypertension, and metabolic syndrome, which may further contribute to sexual problems related to psoriasis [7,18].

Importantly, recent findings have clearly demonstrated that biologic drugs can significantly improve the quality of life of patients with psoriasis. Of note, biologics have been shown to have not only high efficacy but also a favorable safety profile across the entire patient population, including, in particular, vulnerable patient subgroups such as children and the elderly [19,20]. In addition, achieving greater disease control in psoriasis can lead to substantially greater improvement in quality of life and sexual function. According to one study, ustekinumab showed a significantly larger reduction of sexual difficulties in psoriatic patients compared to placebo [21]. Moreover, patients using anti-TNF therapy and anti-IL-17A drugs were noted to have a better quality of sex life [22]. However, some substances, such as methotrexate and acitretin, have been linked to erectile and sexual dysfunction, and their use must be carefully considered among patients who are likely to suffer from a sexual disorder [14]. It has to be underlined that taking into account the frequency and severity of sexual dysfunction in psoriasis patients, as well as the psychological aspect of psoriasis in general, is crucial. One study revealed that as many as 43% of psoriasis patients believe that doctors do not pay enough attention to the problem of sexual disorders. As this problem has been frequently neglected and has not been routinely verified, doctors are strongly encouraged to assess the impact of psoriasis, focusing not only on the visible condition of the skin but also on the impact on mental and sexual well-being [23].

The commonly employed tools for evaluating sexual dysfunction in psoriasis studies, such as the International Index of Erectile Function-5 (IIEF) for men and the Female Sexual Function Index (FSFI) for women, tend to concentrate primarily on assessing genital physiological function. For this reason, these tools may not provide a systemic and comprehensive evaluation of psoriasis’ influence on the sexual life of individuals as it disregards the psychic aspect of sexual intercourse. Similarly, the DLQI, which was implemented to assess quality of life, does not allow us to assess sexual dysfunction accurately as it contains only one question regarding sexual intercourse, which makes it impossible to globally evaluate and determine the degree of sexual dysfunction [24,25].

Nevertheless, bearing the importance of sexual health in mind, one may postulate that an assessment of the sexual dysfunction using dedicated scales should be part of the routine clinical examination implemented for a quick measurement of the severity of psoriasis and identification of patients who require a comprehensive approach and treatment. The aim of our study was to validate the Skin-Related Sexual Life Questionnaire already used by us in our previous studies [3] in the assessment of psoriatic patients. This questionnaire will quickly enable the identification of possible sexual dysfunction and related psychological burdens in patients with psoriasis.

## 2. Methods

### 2.1. Subjects

A total of 109 patients were included in a cross-sectional, questionnaire-based study of sexual dysfunction. Patients were consecutively recruited from women and men appearing for a routine consultation or hospitalization for an exacerbation of psoriasis at the Department of Dermatology in Rzeszow, Poland, between January 2020 and December 2021. All participants were characterized by the following inclusion criteria: consenting adults, speaking the Polish language, and having active psoriatic lesions on the skin. All patients consented to participate in the study. Each participant underwent a careful physical examination, including the assessment of disease severity using the Psoriasis Area and Severity Index (PASI) [26].

### 2.2. Study Design

After providing consent to participate in the study, all participants were asked to fill in a demographics questionnaire. The type of psoriasis was classified based on the appearance and location of the skin lesions. The disease severity was assessed via the PASI scale. Then, all included patients were asked to complete the Dermatology Life Quality Index (DLQI) [27]. Sexual dysfunctions were assessed via the 11-item Skin-Related Sexual Life Questionnaire (SRSLQ) developed and used by our group in previous studies [3,7]. The questionnaire was completed at the time of the first examination and 7 days after inclusion into the study for reassessment to evaluate test-retest reliability. This period was thought to be long enough to answer questions without remembering previous answers, as well as short enough to protect the patient from visible changes in characteristics of psoriasis. Additionally, male participants were asked to complete a 5-item version of the International Index of Erectile Dysfunction (IIEF-5) [28].

### 2.3. Development of Skin-Related Sexual Life Questionnaire

The SRSLQ is designed as an 11-item questionnaire divided into two sections of questions. The first group of 7 question concern psychosocial and emotional problems in men and women with psoriasis related to their sexual life with following possible answers: “never”, “occasionally”, “sometimes”, “often”, and “all the time” (scored from 0 to 4). The second part consists of 4 questions regarding embarrassment and attractiveness level in studied patients with four possibilities to choose: “not at all”, “yes, a little”, “yes, markedly”, and “yes, very much” (scored from 0 to 3). The maximal sum that each patient may obtain for the 11-Item SRSLQ is 40 points, while the minimal is 0.

### 2.4. Statistical Analysis

Statistical analyses were performed using Statistica 13.0 software (Statsoft, Kraków, Poland). The internal consistency of the questionnaire was evaluated via a Cronbach α coefficient. The value of Cronbach α coefficient of at least 0.70 was considered to be good evidence of internal consistency, and the value above 0.90 indicated very good internal consistency [29]. The reproducibility (test–retest reliability) was assessed via comparison of the 2 responses of each participant using the intraclass correlation coefficient (ICC). Adequate reproducibility of the results assessed with the ICC had to be above 0.70 [30]. The normal distribution was verified with the Kolgomorov–Smirnov test. The correlation between individual components and the total score of the questionnaires was calculated with Pearson correlation test. Differences between the first and the second assessment were verified with a Wilcoxon signed-rank test. *p*-values lower than 0.05 were considered statistically significant.

## 3. Results

Validation was based on 109 psoriatic patients (including 45 women and 64 men (1:1.4) with psoriasis aged between 18 and 73 years (mean 48.0 ± 13.4 years). Among all included patients, 91 had plaque-type psoriasis (83.5%), 28 had scalp psoriasis (25.7%), 16 had concomitant nail psoriasis (14.7%), and 14 (12.8%) had palmoplantar pustular psoriasis (Table 1). The average duration of psoriasis at the time of examination was 19.0 ± 12.1 years (range: 1–55 years). The mean psoriasis intensity evaluated with PASI was 17.0 ± 14.9 points (range: 2.0–65.0 points). Participants presented the following localization of psoriatic lesions: the scalp in 80 (73.4%), hands in 62 (56.9%), face in 39 (35.8%) patients, on the nails in 55 (50.5%), and within the genital area in 50 (45.9%) participants.

According to the SRSLQ, 87 (79%) of the psoriatic patients claimed that that their skin condition affected their sexual lives at least occasionally; 41 (37.6%) patients admitted that their sexual activity decreased because of the skin problem at least a little; 24 (22.0%) reported “markedly” and 9 (8.3%) reported “very much” (Table 2 and Table 3).

**Figure 1:**
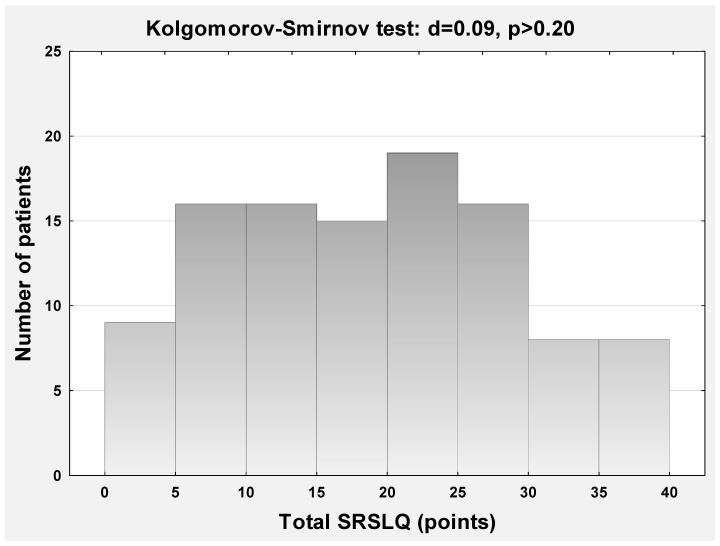
Histogram demonstrating the normal distribution of answers observed in the Skin-Related Sexual Life Questionnaire.

The mean score of the SRSLQ achieved among 107 patients was 19.5 ± 10.2 (range: 1–40). According to Kolgomorov–Smirnov test, the total SRSLQ scoring had normal distribution (d = 0.09; *p* > 0.2) without any bottom or ceiling effect (Figure 1).

Analysis of the internal consistency of SRSLQ showed that all the items correlated with each other. The Cronbach α coefficient value was calculated as 0.94, indicating very good internal consistency of the questionnaire. Furthermore, significant correlations were observed between answers to single questions and the SRSLQ total scoring, confirming the good internal consistency of the instrument (Table 4).

The reproducibility of the SRSLQ was assessed on a group of 35 participants. The intraclass correlation coefficient (ICC) between the two SRSLQ amounted to 0.96. Significant differences were observed only in two questions, although the differences were small. In Question 5, the second assessment demonstrated slightly lower values, while in Question 7, they were minimally higher. Nevertheless, the total scoring at day 0 and day 7 were almost identical (Table 5).

**Figure 2:**
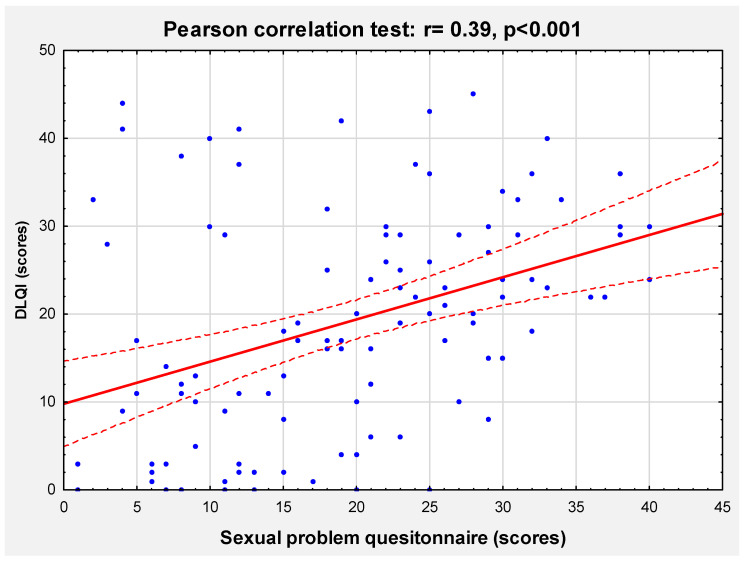
Correlation between the Skin-Related Sexual Life Questionnaire and Dermatology Life Quality Index (DLQI), confirming convergent validity of the new scale.

There was a significant correlation between the total score of SRSLQ and DLQI (r = 0.39; *p* < 0.001) (Figure 2), confirming the convergent validity of the new instrument. Interestingly, no correlation was found between DLQI and erectile problems assessed via the IIEF-5 (r = 0.01; *p* = 0.99).

## 4. Discussion

The problem of the stigmatization of patients affected by skin diseases has received much attention recently. To date, many studies have been conducted highlighting the role of stigma in the occurrence of significant limitations in the social and psychological life of patients with psoriasis [4,31]. Previous studies have highlighted a notably elevated occurrence of diverse psychological issues among this group. People suffering from psoriasis encounter rejection, live with a constant feeling of shame, and often give up activities in different spheres of life due to the skin condition, which significantly reduces their quality of life. Moreover, the impact on patients’ quality of life is often higher than in other chronic diseases [32]. Psoriasis is commonly a source of fear in the social environment and mistaken for a contagious disease or the result of poor personal hygiene [33]. Additionally, psoriatic patients, according to Picardi et al., tend to report lower levels of perceived social support in comparison to those with other conditions [34].

One study showed that 44.7% of men experienced rejection because of psoriasis. Moreover, the feeling of being unattractive or embarrassed during psoriasis exacerbation can lead to low self-confidence and erectile dysfunction [23]. Our study was consistent with these results. According to the survey, more than 90% of the patients felt at least slightly unattractive due to psoriasis. Furthermore, many participants (over 50%) reported occasionally abstaining from sexual encounters due to their psoriasis condition [3].

Since physical appearance is an element of self-confidence and plays an important role in interpersonal relationships, the presence of psoriatic lesions can impede sexual initiation [24,35]. It was discovered that the part of the body affected by psoriasis may have an impact on the occurrence of sexual dysfunction [15].

In our earlier study [3], we noted a significant correlation between the level of sexual dysfunction and the location of psoriasis, particularly in the genital area (*p* = 0.01), on the face (*p* = 0.03), and on the hands (*p* = 0.05). Accordingly, we have shown that visible scaly skin lesions are clearly associated with lower self-esteem and significantly reduce sexual activity among psoriasis patients [3].

In another study, psychological stress and high PASI were also assessed as independent risk factors for sexual dysfunction in women with psoriasis. The suggested relationship between the severity of psoriasis and sexual dysfunction was proven in studies, where patients who achieved a higher improvement of skin lesions (assessed via PASI) experienced a greater reduction in sexual dysfunction [21].

According to a previously conducted study, individuals who perceive their psoriasis as severe experienced significantly more sexual problems, with an average score of 22.1 ± 9.9 points, in contrast to those who considered their psoriasis to be moderate (average score: 16.2 ± 9.1 points) or mild [3]. It may show that the perception of the severity of the disease is a very individual matter, depending on beliefs, personal experiences, and one’s environment. However, the psychosocial impact of psoriasis is often not adequately represented in physicians’ objective assessments of the condition’s severity [36]. Consequently, the overall severity of the disease may thus be underestimated.

Since the problem of sexual dysfunction in psoriasis has been demonstrated in both our own and the previously mentioned studies, there is a need for tools with which to assess the severity of these problems in order to quickly identify affected patients requiring intensified psoriasis therapy and an overall holistic approach to treating comorbid disorders.

Increased awareness of comorbidities in psoriasis implies the need for more accurate and reliable measurements. However, assessing sexual dysfunction can be difficult due to the different subjective feelings, the duration of dysfunction, and the shame felt by patients. To date, the IIEF-5 questionnaire in men and the Female Sexual Function Index (FSFI) questionnaire in women, as well as the Dermatology Life Quality Index, have been used to assess sexual dysfunction in routine clinical practice in patients with psoriasis [14,37]. However, there are some limitations to using these questionnaires; e.g., DLQI was not specifically designed for this purpose; it may omit descriptions of sexual dysfunction that the patient does not consider to be directly related to psoriasis but rather treats it as a comorbid condition [2]. The IIEF-5 and FSFI questionnaires, on the other hand, cover a short period of time (the last 4 weeks), are very detailed, and should not be used with patients who are not currently sexually active [38].

Here, we proposed a new instrument for assessing sexual dysfunction related to skin conditions. In our view, the visibility of skin lesions is a key factor influencing feelings of stigma and related burdens, including sexual dysfunction. In our previous study [3], we confirmed that skin condition is often a determinant of a patient’s subjective perception of disease severity and show a positive relationship with the quality of life. Moreover, we believe that our proposed assessment tool may be useful in evaluating patients with other skin dermatoses, such as atopic dermatitis or hidradenitis suppurativa, and will contribute to a better understanding of patients’ suffering, as well as emphasize the role of a holistic approach.

It should be mentioned that our study also contains several limitations. One of them is the rather small group of patients on which reproducibility could be assessed, and we hope to continue the validation process in the future as assessing the validity of any new tool is always a lengthy process. Additionally, the skin lesions may differ from those present when the first questionnaire was filled out due to the therapy implemented. Moreover, the impact of psoriasis may depend on the disease subtype, a relationship which has not been studied in depth in the current report. In our recent study on pruritus in psoriasis, we have found some differences between the level of quality of life impairment as assessed with DLQI and the subtype of psoriasis, which may also have an influence on the degree of sexual satisfaction [39]. However, the extent of the skin involvement does not necessarily correlate with the impairment of the patient’s well-being and sexual problems, and even relatively limited lesions, e.g., psoriasis involving only nails, may cause significant suffering and stigmatization, a phenomenon that needs further investigation [40]. Nevertheless, despite the aforementioned limitations, we still consider the new tool for assessing sexual dysfunction to be valuable and worth further testing in future clinical trials.

## 5. Summary

The ongoing search for and interest in developing new therapies for a wide range of dermatologic conditions creates a need to explore and validate new instruments with which to comprehensively assess the disease and its various comorbidities. We would like to emphasize that the 11-item Skin-Related Sexual Life Questionnaire has proven to be an effective tool for accurately assessing skin-related sexual dysfunction, with high convergent validity, good test-retest reproducibility, and satisfactory convergent validity. We believe that the availability of this questionnaire will contribute to a better understanding of the suffering of patients affected by dermatological conditions and stimulate further research on stigma and sexual dysfunction among patients with various dermatoses. All of the above results suggest that the 11-item SRSQL fulfilled the criteria for high-standard instruments and may be used as a routine assessment tool in clinical practice among dermatologists.

## Figures and Tables

**Table 1:** Patients’ characteristics (max—maximum; min—minimum; SD—standard deviation).

Age (years) [mean ± standard deviation (min–max)]	48.0 ± 13.4 (18–76)
Psoriasis duration (years) [mean ± standard deviation (min–max)]	19.0 ± 12.1 (1–55)
Specific location of skin lesions	
- Face	39 (35.8%)
- Hands	62 (56.9%)
- Genital area	50 (45.9%)
- Scalp	80 (73.4%)
- Nails	55 (50.5%)
Type of psoriasis *	
- Plaque-type psoriasis	91 (83.5%)
- Psoriatic arthritis	18 (16.5%)
- Scalp psoriasis	28 (25.7%)
- Nail psoriasis	16 (14.7%)
- Palmoplantar pustular psoriasis	14 (12.8%)
Patient’s assessment of psoriasis severity	
- Mild	8 (7.3%)
- Moderate	32 (29.4%)
- Severe	69 (63.3%)

* More than one psoriasis subtype could be present.

**Table 2:** Results on psychosocial and emotional problems in men and women with psoriasis related to their sexual lives—part one.

	Never	Occasionally	Sometimes	Often	All the Time	Missing Data
Did your skin condition ever influence your sexual life?	19 (17.4%)	20 (18.3%)	21 (19.3%)	32 (29.4%)	14 (12.8%)	3 (2.8%)
Do you think that other people considered your skin problem as contagious disease?	9 (8.3%)	11 (10.1%)	35 (32.1%)	33 (30.3%)	19 (17.4%)	2 (1.8%)
Do you avoid social contacts due to your skin problem?	24 (22.0%)	24 (22.0%)	22 (20.2%)	25 (22.9%)	12 (11.0%)	2 (1.8%)
Do you avoid sexual intercourses due to your skin problem?	24 (22.0%)	19 (17.4%)	30 (27.5%)	24 (22.0%)	8 (7.3%)	4 (3.7%)
Do you feel ashamed of your skin when you are together with your sexual partner?	26 (23.9%)	16 (14.7%)	20 (18.3%)	31 (28.4%)	12 (11.0%)	4 (3.7%)
Do you experience rejection due to your skin condition?	42 (38.5%)	15 (13.8%)	30 (27.5%)	13 (11.9%)	6 (5.5%)	3 (2.8%)
Are you stressed before the sexual intercourse due to your skin condition?	27 (24.8%)	22 (20.2%)	17 (15.6%)	25 (22.9%)	14 (12.8%)	4 (3.7%)

**Table 3:** Results regarding embarrassment and attractiveness level in studied patients.

	Not at All	Yes, a Little	Yes, Markedly	Yes, Very Much	Missing Data
Do you feel unattractive due to your skin disease?	6 (5.5%)	31(28.4%)	34 (31.2%)	36 (33.0%)	2 (1.8%)
Do you feel embarrassed when the skin lesions are present on visible body areas?	5 (4.6%)	33 (30.3%)	30 (27.5%)	39 (35.8%)	2 (1.8%)
Do you feel embarrassed when the skin lesions are present on genital area?	15 (13.8%)	22 (20.2%)	38 (34.9%)	30 (27.5%)	4 (3.7%)
Did your sexual activity decrease due to skin problem?	31 (28.4%)	41 (37.6%)	24 (22.0%)	9 (8.3%)	4 (3.7%)

**Table 4:** The correlation coefficients between the answers to each question and the total score of the Skin-Related Sexual Life Questionnaire (*p* < 0.001 for all comparisons).

	Q1	Q2	Q3	Q4	Q5	Q6	Q7	Q8	Q9	Q10	Q11
Q1	-										
Q2	0.48	-									
Q3	0.46	0.77	-								
Q4	0.55	0.70	0.73	-							
Q5	0.42	0.58	0.55	0.51	-						
Q6	0.58	0.54	0.51	0.53	0.58	-					
Q7	0.65	0.61	0.58	0.63	0.48	0.64	-				
Q8	0.72	0.57	0.51	0.58	0.45	0.65	0.71	-			
Q9	0.62	0.45	0.49	0.52	0.35	0.54	0.58	0.68	-		
Q10	0.56	0.51	0.53	0.51	0.50	0.59	0.71	0.58	0.54	-	
Q11	0.68	0.52	0.53	0.56	0.47	0.61	0.77	0.76	0.67	0.70	-
Total	0.79	0.75	0.74	0.76	0.66	0.78	0.86	0.84	0.74	0.77	0.85

**Table 5:** Comparison of each item and total questionnaire scoring at day 0 and day 7 (results demonstrated as means and standard deviations).

	Day 0	Day 7	*p* (Paired Test T)
Question 1	2.4 ± 1.3	2.6 ± 1.2	0.06
Question 2	2.1 ± 0.9	2.0 ± 0.9	0.53
Question 3	2.0 ± 1.0	2.1 ± 0.9	0.37
Question 4	1.8 ± 1.0	1.9 ± 1.0	0.21
Question 5	2.6 ± 1.1	2.4 ± 1.1	0.01
Question 6	2.2 ± 1.3	2.1 ± 1.3	0.54
Question 7	2.0 ± 1.1	2.1 ± 1.2	0.02
Question 8	2.3 ± 1.3	2.2 ± 1.3	0.26
Question 9	1.3 ± 0.9	1.3 ± 1.0	0.66
Question 10	1.6 ± 1.3	1.6 ± 1.2	0.66
Question 11	2.2 ± 1.4	2.1 ± 1.4	0.41
Total scoring	22.1 ± 10.0	22.1 ± 10.0	0.95

## Data Availability

The data presented in this study are available on request from the corresponding author. The data are not publicly available due to General Data Protection Regulation—sensitive patient data.
